# Neurocognitive and Synaptic Potentiation Deficits Are Mitigated by Inhibition of HIF1a Signaling following Intermittent Hypoxia in Rodents

**DOI:** 10.1523/ENEURO.0449-20.2020

**Published:** 2020-11-24

**Authors:** Rosalind S.E. Carney

## Abstract

**Highlighted Research Paper:** A HIF1a-Dependent Pro-Oxidant State Disrupts Synaptic Plasticity and Impairs Spatial Memory in Response to Intermittent Hypoxia. Alejandra Arias-Cavieres, Maggie A. Khuu, Chinwendu U. Nwakudu, Jasmine E. Barnard, Gokhan Dalgin and Alfredo J. Garcia III

Sleep apnea is a condition that affects ∼18 million adults in the United States (National Sleep Foundation, 2020). Sleep apnea, which also affects children, can result in impairment of cognitive functions, such as learning and memory, attention, and emotional dysregulation, including depression (Beebe and Gozal, 2002; Schröder and O'Hara, 2005; Jackson et al., 2011; Wallace and Bucks, 2013; Varga et al., 2014; Gildeh et al., 2016; Devita et al., 2017a,b; Leng et al., 2017). Brain imaging studies in humans have shown that sleep apnea negatively affects multiple brain regions, including the hippocampus, which is involved in learning and memory processes (Sforza et al., 2016; Cha et al., 2017; Macey et al., 2018; Song et al., 2018). The neurocognitive defects associated with sleep apnea result from the periods of intermittent hypoxia (IH), caused by repeated cycles of disrupted breathing during sleep. The neural substrates of IH-induced cognitive dysfunction have been examined in rodents using intermittent or sustained IH-exposure paradigms. IH, rather than sustained hypoxia, induces the cardiorespiratory responses that best mimic sleep apnea (Peng and Prabhakar, 2004). In rodents, IH has been shown to impair spatial learning in rats (Row et al., 2002; Gozal et al., 2003), weaken synaptic plasticity (Goldbart et al., 2003; Payne et al., 2004; Xie et al., 2010; Zhang et al., 2012; Wall et al., 2014; Khuu et al., 2019), and increase oxidative stress (Nair et al., 2011; Chou et al., 2013). An IH-exposure-dependent shift toward a pro-oxidant state has been linked to upstream signaling of the transcriptional activator hypoxia-inducible factor 1a (HIF1a) *in vitro* and in *HIF1a* heterozygous knock-out (*HIF1a^+/–^*) mice (Peng et al., 2006). HIF1a expression was known to be upregulated in the cerebrum following IH (Peng et al., 2006), and HIF1a signaling can also induce pro-survival processes. Therefore, it was unknown whether HIF1a expression is altered specifically in the hippocampus following IH exposure, and if so, whether HIF1a-dependent signaling is responsible for neurocognitive defects associated with sleep apnea. In their *eNeuro* publication, Arias-Cavieres and colleagues examined the effects on IH on neurocognitive function, hippocampal synaptic and molecular physiology in wild-type (WT) mice and heterozygous *HIF1a^+/–^* knock-out (*HIF1a^+/–^*) mice.

Adult male and female WT and *HIF1a^+/–^* (Iyer et al., 1998; Peng et al., 2006) mice were used in the study as homozygous knock-outs for *Hif1a* are embryonic lethal. IH exposure occurred for 10 consecutive days (IH_10_) and included hypoxic cycles of 100% N_2_ air flow into a chamber. Each hypoxic cycle lasted for 1 min, followed by an air break, rendering a total of 80 hypoxic cycles during a daily 8-h period.

The authors first confirmed whether IH exposure alters HIF1a subcellular localization in the hippocampus. Hippocampal tissue was processed for Western blot analysis to quantify nuclear levels of HIF1a in WT mice unexposed (control) or exposed to IH_10_ (WT IH_10_). Following IH_10_, nuclear levels of HIF1a were two times greater compared with the control group. This finding shows that IH results in increased cytoplasmic-to-nuclear translocation of HIF1a within the hippocampus, positioning HIF1a in the nucleus to potentially bind to regulatory elements of hypoxic-responsive genes.

As IH altered HIF1a expression in the hippocampus, the authors determined whether spatial learning and memory were affected by IH exposure. The Barnes maze is a circular arena that contains 20 equidistant exit holes at the perimeter. During training and probe trials, each mouse was individually placed in the middle of the arena to eliminate any directional bias within the arena; no distinct visual cues were present outside of the arena. The authors designed exit number 20 as the target exit throughout the experiments. During training, all exit holes except Exit 20 were closed. At Exit 20, there was a ramp that led into a small box external to the arena. For three consecutive days, each mouse underwent a daily 6-min trial in which the latency and distance from placement into the maze to initial entry into the exit zone at Exit 20 were recorded. Upon failure to locate the target exit, the mouse was guided toward Exit 20, and a latency of 6 min was recorded. Whereas the training period assessed learning capability, the probe trial (day 4), served as a measure of memory. During the probe trial, all 20 exit holes were closed. The latency and distance to initial entry to the exit zone at Exit 20 and overall time spent at each exit were recorded.

Arias-Cavieres and colleagues found that latency and distance to initial entry to the exit zone were similar between control and IH_10_ WT mice, which demonstrated that IH exposure did not result in spatial learning or locomotor deficits. However, in the probe trial, WT IH_10_ mice exhibited significantly longer initial latency and distance to the entry zone at Exit 20 compared with control mice (Fig. 1*A*,*B*). To verify that the initial exit that each mouse visited accurately reflected overall recall of the target exit, the authors created a heat map for each experimental group of the average probability of entry to each exit zone during the probe trial. In the heat map, a “hot” (red) region indicates a higher average of repeated visits to an exit zone than a “cold” (blue) region. The heat maps showed that control mice were more likely to repeatedly enter the exit zone at Exit 20 than WT IH_10_ mice (Fig. 1*C*,*D*). Instead, WT IH_10_ mice could not discern where the target exit zone, Exit 20, was relative to the other exits. These results indicate that IH exposure impaired the ability of WT mice to recall the position of the target exit.

**Figure 1.**
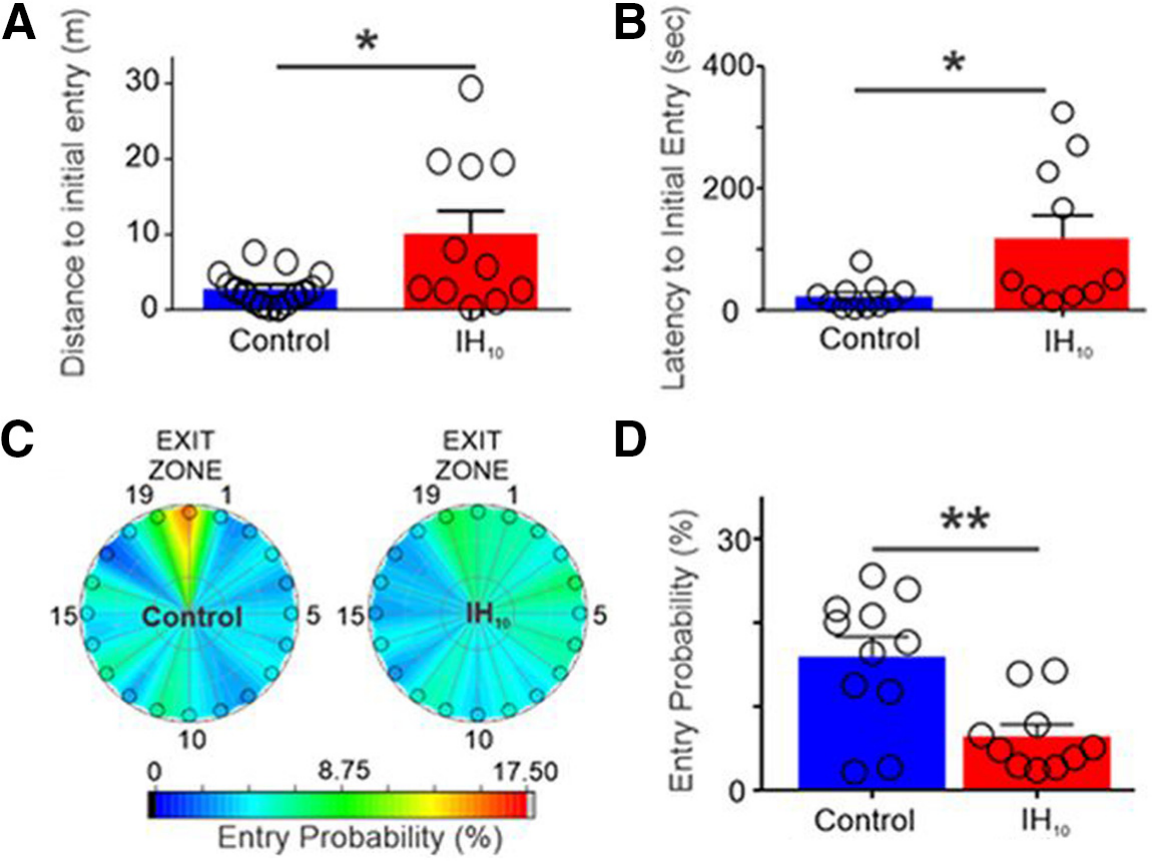
Ten days of IH disrupts Barnes maze performance in WT mice. ***A***, During the probe trial, the distance traveled to initially enter the exit zone was shorter in control mice compared with WT IH_10_ mice. ***B***, Latency to initial entry was also shorter in control mice. ***C***, Heat maps of the mean entry probability across all false exits (1–19) and Exit 20 during probe trial for the control and WT IH_10_ mice. ***D***, Comparison of entry probability into the exit zone during the probe trial reveals that control mice had a greater probability of entering the exit zone when compared with WT IH_10_ mice. (Adapted from Figure 1 in Arias-Cavieres *et al*., 2020.)

To determine whether the IH-induced memory deficit was dependent on HIF1a signaling, the same training and probe trial experiments were performed using 0-*HIF1a^+/−^* (no IH exposure) and 10-*HIF1a^+/−^* (10 d of IH exposure) mice. Both 0-*HIF1a^+/−^* and 10-*HIF1a^+/−^*mice exhibited comparable initial distance and latency to Exit 20 during training (Fig. 2*A*,*B*), and similar entry zone probabilities during the probe trial (Fig. 2*C*,*D*). In contrast to WT mice, in which IH exposure resulted in increased nuclear localization of HIF1a in hippocampal tissue, nuclear versus cytoplasmic levels of HIF1a were unaffected by IH exposure in *HIF1a^+/−^* mice. Thus far, the results of the HIF1a localization and behavioral analyses indicated that IH exposure was associated with a neurocognitive memory defect in mice that had high nuclear expression levels of HIF1a.

**Figure 2.**
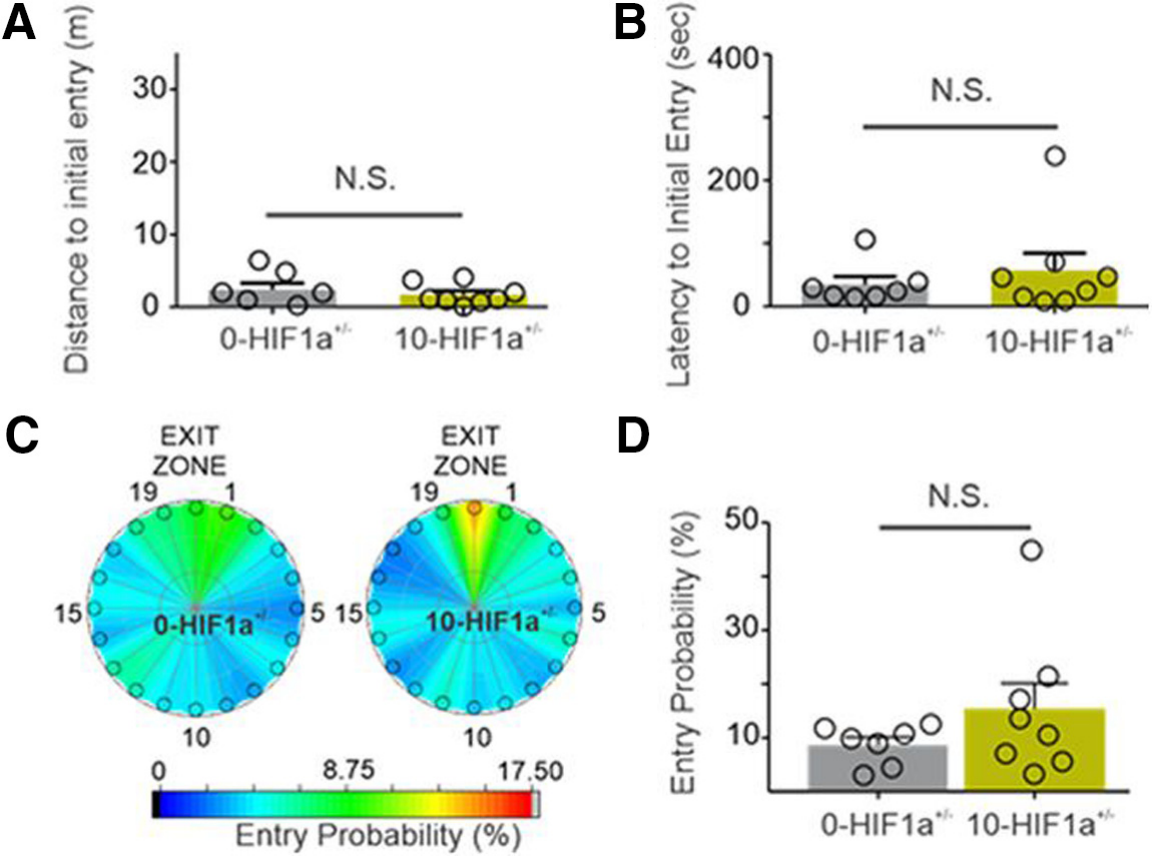
Ten days of IH does not affect Barnes maze performance in *HIF1a^+/−^* mice. ***A***, In *HIF1a^+/−^* mice, the distance initial to initial entry into the exit zone (Exit 20) was similar between 0-*HIF1a^+/−^* and 10-*HIF1a^+/−^* mice. ***B***, Latency to initial entry into the exit zone (Exit 20) during the probe trial was similar between 0-*HIF1a^+/−^* and 10-*HIF1a^+/−^* mice. ***C***, Heat maps of the mean entry probability into all zones during the probe trial for 0-*HIF1a^+/−^* and 10-*HIF1a^+/−^* mice. Entry probability was similar between 0-*HIF1a^+/−^* and 10-*HIF1a^+/−^* mice; *p <* *0.05, **p <* *0.01; N.S., not significant. (Adapted from Figure 1 in Arias-Cavieres *et al*., 2020.)

Once a behavioral deficit associated with HIF1a signaling had been established, the authors examined whether IH exposure affected long-term potentiation, a neural substrate of both learning and memory processes. High-frequency stimulation (HFS) was used to evoke LTP (LTP_HFS_) in the CA1 area of hippocampal slices isolated from control and WT IH_10_ mice. In control mice, LTP_HFS_ was attenuated in the presence of AP5, an NMDA receptor (NMDAr) antagonist. In slices from WT IH_10_ mice, LTP was attenuated by HFS both in the absence or presence of AP5. LTP magnitude was weaker in WT IH_10_ mice compared with control mice, which, combined with the lack of effect of AP5, demonstrates that IH exposure causes an NMDAr-mediated suppression of LTP. In contrast, in the absence of IH exposure, LTP_HFS_ responses comprise both NMDAr-dependent and NMDAr-independent mechanisms. However, following IH_10_, the NMDAr-dependent component of LTP in WT mice was no longer sensitive to AP5, suggesting that an NMDAr-independent mechanism was predominantly responsible for LTP when WT mice had been exposed to IH. In 0-*HIF1a^+/−^* and 10-*HIF1a^+/−^*mice, HFS evoked a similar magnitude of LTP. Overall, these findings suggest that hippocampal synaptic physiology is affected by IH in mice with high nuclear expression of HIF1a and that suppressed synaptic potentiation may be more relevant to NMDAr-dependent, rather than NMDAr-independent, LTP mechanisms.

To further examine the NMDAr-dependent LTP response following IH, the authors performed a separate set of experiments using theta-burst stimulation (TBS) to evoke LTP (LTP_TBS_). LTP_TBS_ is only NMDAr dependent; thereby, this electrophysiological approach circumvents the NMDAr-independent LTP mechanisms. In control mice, LTP_TBS_ was evoked but was blocked by AP5 (Fig. 3*A*). In contrast, LTP_TBS_ was not evoked in WT IH_10_ mice showing that IH affected NMDAr-dependent synaptic potentiation (Fig. 3*B*,*C*). The effect of IH on LTP_TBS_ was associated with HIF1a expression, as 0-*HIF1a^+/−^* and 10-*HIF1a^+/−^*mice showed a comparable magnitude of LTP_TBS_, and AP5 blocked LTP_TBS_ in 10-*HIF1a^+/−^*mice (Fig. 3*D*,*E*). Collectively, the LTP_TBS_ results suggested that IH affects the NMDAr physiology of LTP in a HIF1a-dependent manner. This conclusion was further supported by additional experiments that showed that the expression of GluN1, an obligatory NMDAr subunit, was reduced in WT IH_10_ mice compared with controls. GluN1 expression levels in 0-*HIF1a^+/−^* and 10-*HIF1a^+/−^*mice were similar to the expression levels in control mice, supporting a HIF1a-dependent role of the effect of IH on NMDAr physiology. IH did not impact expression levels of a postsynaptic density marker, suggesting that the effect of IH was specific to the GluN1 subunit rather than a broad dysregulation of glutamatergic synapse components.

**Figure 3.**
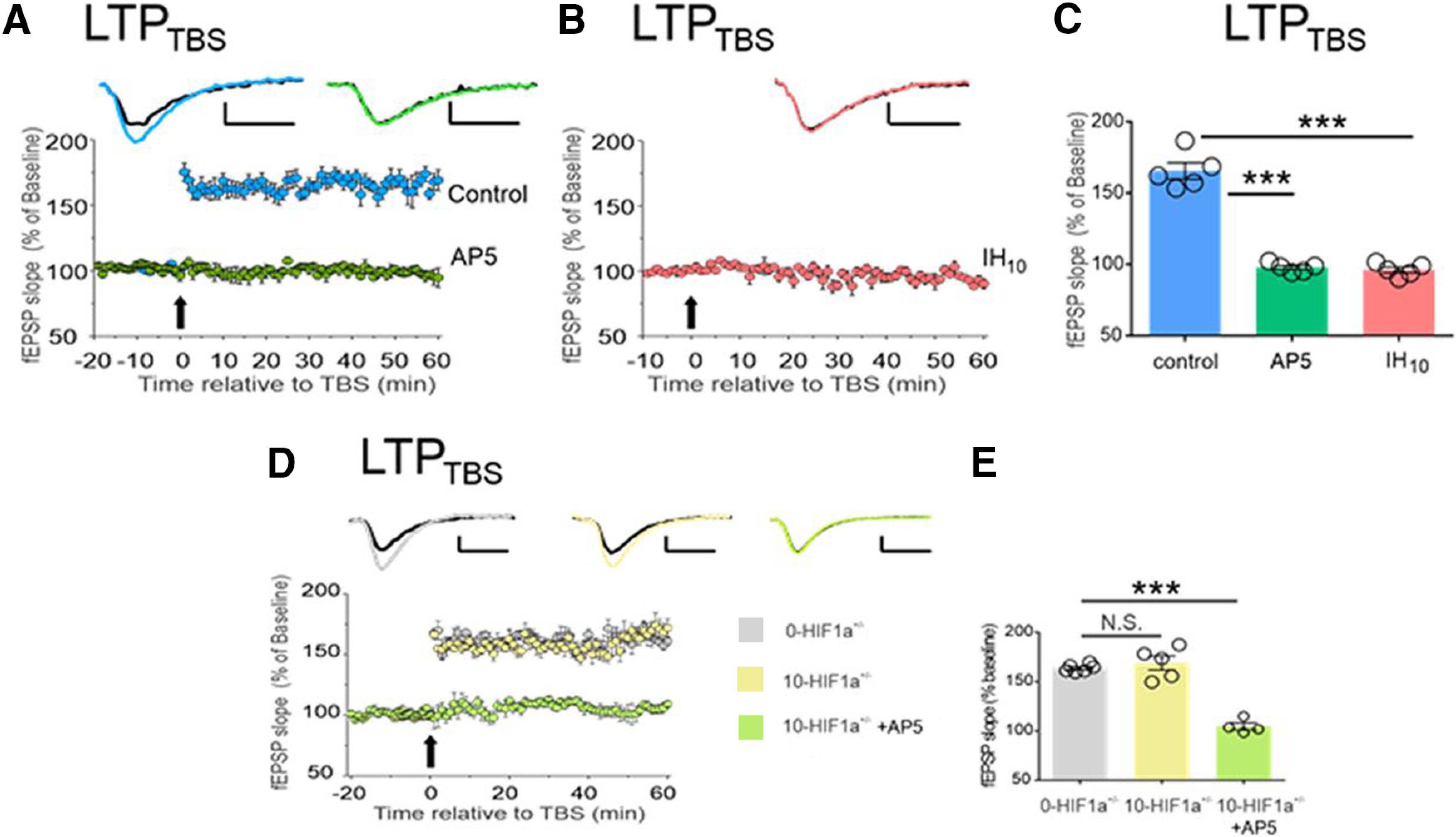
IH suppresses NMDAr-dependent synaptic potentiation in WT hippocampal slices, but NMDAr-dependent LTP is unaffected by IH in the hippocampal slices from *HIF1a^+/−^*mice. ***A***, LTP_TBS_ is readily evoked in control (light blue) and is completely blocked by AP5 (light green). ***B***, LTP_TBS_ is present following IH (pink). ***C***, *Post hoc* comparison of LTP_TBS_ magnitude (60 min following TBS) show significant effects of AP5 or IH_10_ compared with the control condition. ***D***, LTP_TBS_ was evoked in 0-*HIF1a^+/−^* (light gray), 10-*HIF1a^+/−^* (light yellow), and 10-*HIF1a^+/−^* + AP5 (light green) groups. No difference was found when comparing LTP_TBS_ magnitude of 0-*HIF1a^+/−^* and 10-*HIF1a^+/−^* mice. Representative traces illustrate baseline (black) and 60 min following HFS (colored trace). Scale bars: 0.2 mV/10 ms. In experiments using AP5, electrophysiological recordings began at 20 min before eliciting LTP (i.e., *t* = −20), while AP5 was applied 10 min before eliciting LTP (i.e., *t* = −10). For all experiments, the arrow represents the electric protocol (TBS); ***p <* *0.01; N.S., *p *>* *0.05. (Adapted from Figure 2 in Arias-Cavieres *et al*., 2020.)

Thus far, the experiments supported an IH-induced, HIF1a-dependent neurocognitive defect and molecular defects specific to synaptic potentiation. Next, Arias-Cavieres and colleagues examined changes in redox state in the experimental groups. HIF1a regulates the expression of NOX4 (Diebold et al., 2010), an NADPH oxidase that produces reactive oxygen species. Western blot analysis was used to determined NOX4 levels relative to the housekeeping gene GADPH in hippocampal tissue isolated from control, WT IH_10_, 0-*HIF1a^+/−^*, and 10-*HIF1a^+/−^* mice. WT IH_10_ mice had higher NOX4 expression levels compared with controls; expression in 0-*HIF1a^+/−^* and 10-*HIF1a^+/−^* mice was similar to controls (Fig. 4*A*). These results show that IH exposure results in an intracellular shift to a pro-oxidant state that is dependent on HIF1a signaling.

**Figure 4.**
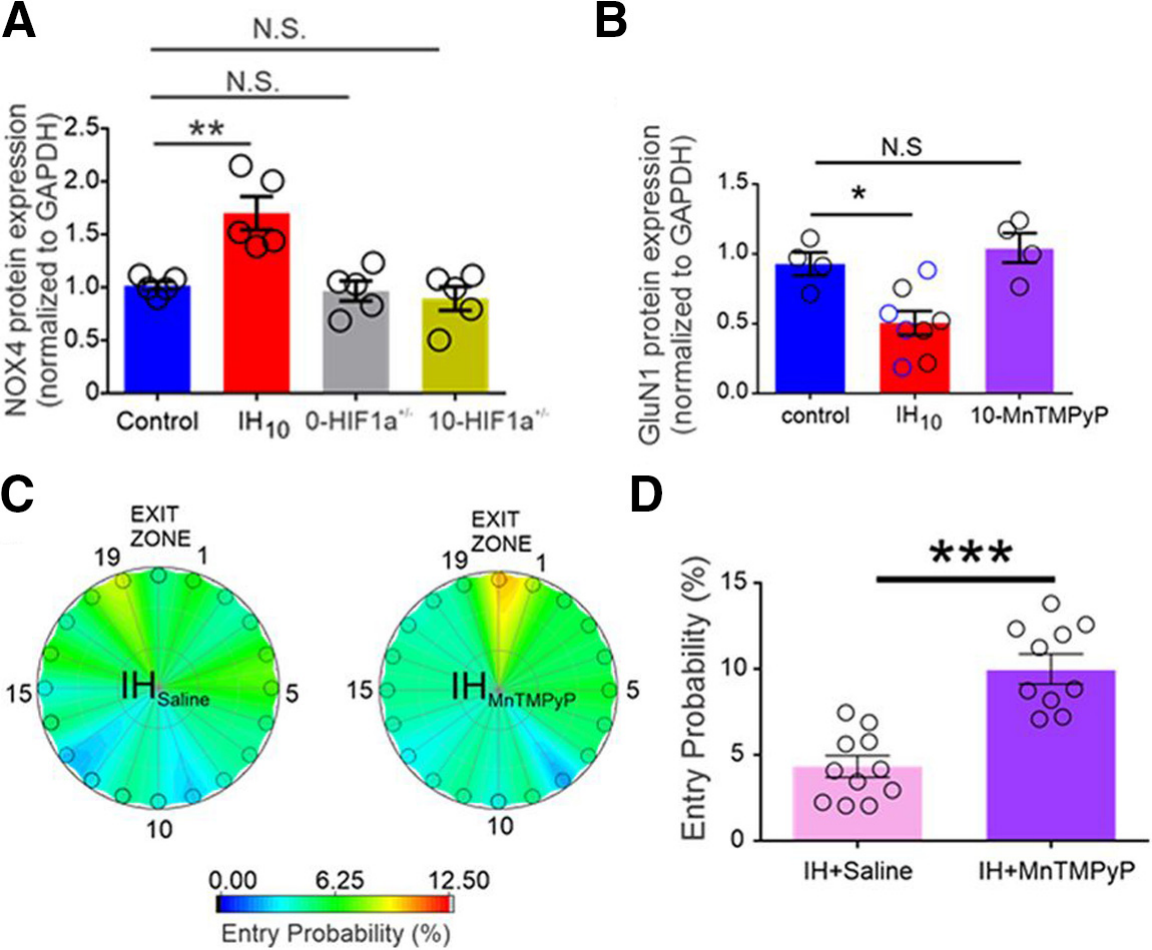
Effects of IH on NOX4 expression and mitigation of IH-dependent effects on GluN1 expression and Barnes maze performance deficits by antioxidant treatment. ***A***, Comparison of the prooxidant enzyme NOX4 expression levels in hippocampal homogenates from control, WT IH_10_, 0-*HIF1a^+/−^*, and 10-*HIF1a^+/−^* mice reveals that NOX4 is increased in WT IH_10_ but not elevated in either 0-*HIF1a^+/−^* or 10-*HIF1a^+/−^* mice. ***B***, Normalized GluN1 protein expression was examined in control, WT IH_10_, IH_Saline_, and IH_MnTMPyP_ mice. No difference in GluN1 expression was evident between IH_10_ (open black circles in IH_10_ label) and IH_Saline_ (open blue circles in IH_10_ label); therefore, the two groups were merged into the IH_10_ label for comparisons to control. Comparisons revealed that GluN1 was reduced only in IH_10_ mice and unchanged in IH_MnTMPyP_ mice. ***C***, Heat maps of the mean entry probability across all false exits (1–19) and the exit zone (Exit 20) during the probe trial for IH_Saline_ and IH_MnTMPyP_ treatments. ***D***, Comparison of entry probability into the exit zone during the probe trial reveals that IH_MnTMPyP_ mice had a greater probability of entering the exit zone when compared with IH_Saline_ mice; *p <* *0.05, **p <* *0.01, ***p <* *0.001; N.S., *p *>* *0.05. (Adapted from Figures 4 and 5 in Arias-Cavieres *et al*., 2020.)

To investigate whether a pro-oxidant state was associated with the dysregulation of GluN1 subunit expression, the authors measured GluN1 levels normalized to GADPH in four experimental groups of WT mice. In addition to control and WT IH_10_ mice, WT mice exposed to 10 d of IH exposure were administered daily injections of saline (IH_saline_) or the superoxide anion scavenger MnTMPyP (IH_MnTMPyP_). IH exposure led to reduced GluN1 expression levels in the WT IH_10_ groups and IH_saline_ groups; however, GluN1 levels were similar between the control and IH_MnTMPyP_ groups (Fig. 4*B*). These observations suggest that inhibiting a pro-oxidant state prevented the molecular dysregulation of the NMDAr.

To determine whether MnTMPyP could rescue the neurocognitive deficit exhibited by IH_10_ mice, Arias-Cavieres and colleagues repeated the LTP_TBS_ and Barnes Maze experiments, including the IH_MnTMPyP_ group. LTP_TBS_ was evoked in hippocampal slices isolated from IH_MnTMPyP_ mice showing that inhibition of the pro-oxidant state rescued the IH-induced phenotype. In the Barnes maze probe trial, IH_saline_ and IH_MnTMPyP_ mice exhibited similar initial distance and latency to Exit 20. However, the heat map results revealed that the entry probability into the exit zone at Exit 20 was higher in IH_MnTMPyP_ mice compared with IH_saline_ mice (Fig. 4*C*,*D*). This observation indicates that recall of the target exit location was more evident by the overall visits to Exit 20 by IH_MnTMPyP_ mice compared with IH_saline_ mice. Therefore, rescue of the pro-oxidant phenotype mitigated the neurocognitive deficit that is normally induced by IH exposure.

This publication is an advance in the field because it provides evidence that HIF1a signaling mediates the adverse effects of IH exposure. HIF1a signaling is upstream of molecular aberrations that are linked to neurocognitive defects, positioning HIF1a signaling as a good target for potential therapies for sleep apnea. The neurocognitive defects associated with sleep apnea are often comorbid in other neurological disorders such as Alzheimer’s disease and Parkinson’s disease. Therefore, it is important to mitigate the neurocognitive defects of sleep apnea, in particular in individuals who have increased susceptibility to neurological disorders in which cognitive function is impaired.
